# Depression and anxiety as predictors of performance status and life satisfaction in older adult neurological patients: a cross-sectional cohort study

**DOI:** 10.3389/fpsyt.2024.1412747

**Published:** 2024-05-20

**Authors:** Mariola Głowacka, Natalia Przybyła, Marzena Humańska, Maciej Kornatowski

**Affiliations:** ^1^ Institute of Nursing, Department of Integrated Medical Care, Faculty of Health Sciences, Collegium Medicum, The Mazovian University in Płock, Płock, Poland; ^2^ Department of Applied Neurocognitivistics, Pomeranian Medical University in Szczecin, Szczecin, Poland; ^3^ Faculty of Health Sciences, Ludwik Rydygier Collegium Medicum, Nicolaus Copernicus University in Toruń, Bydgoszcz, Poland; ^4^ Medical Department, Collegium Medicum, The Mazovian University in Płock, Płock, Poland

**Keywords:** depression, anxiety, satisfaction with life, performance status, stroke, neurological disorders

## Abstract

Neurological disorders are one of the leading causes of disability globally. Studies emphasise that the course and effectiveness of rehabilitation interventions may be influenced by emotional factors. The aim of the present study was to assess the prevalence of depressive and anxiety symptoms in patients with neurological disorders and examine whether depression and tendency to respond with anxiety are predictors of disability in these patients. The study included 229 individuals with neurological disorders aged over 55. Our findings show that stroke patients are more likely to experience depressive symptoms and are more likely to display trait anxiety as compared with patients with other neurological disorders. Advanced age, female sex, low level of education and the presence of trait anxiety are associated with a higher severity of depressive symptoms. Stroke survivors have significantly poorer ECOG performance scores and are more likely to be incapable for work. Both depression and trait anxiety are significant predictors of neurological patients’ performance status, and the presence of depressive symptoms is a determinant of these patients’ level of satisfaction with life. An understanding of psychological risk factors for poorer performance status in individuals with neurological disorders will make it possible to plan prophylactic interventions in order to minimise negative health impacts and facilitate access to mental health facilities.

## 1 Introduction

Neurological disorders, which encompass a range of conditions affecting the brain and nervous system, are a major global health concern. They are the leading cause of disability and the second leading cause of death worldwide. The prevalence of these disorders is increasing, particularly due to the aging population, and they significantly impact low-income and middle-income countries. This trend highlights the urgent need for effective prevention and management strategies to address the growing challenge of neurological disorders globally (1)​. Moreover, the recent COVID-19 pandemic has further complicated the mental health landscape, exacerbating symptoms of depression and anxiety, which are critical factors influencing the management and outcomes of neurological conditions (2, 3). These disorders affect almost all areas of patients’ functioning, including in particular the musculoskeletal system, visual perception as well as cognitive and emotional processes (4–6). Patients with disability due to a neurological disorder withdraw from their previous social and professional lives, have a lower perceived quality of life and require continuous treatment due to their persisting impairments. As a result, the number of health services provided and socioeconomic costs increase (7, 8). Mood and anxiety disorders are one of the causes of chronic disability, especially in older adult individuals. Older adult people are particularly at risk of neurological disorders, notably cerebrovascular disorders, due to the presence of significant risk factors, such as age and sex, and comorbidities (9, 10). Mental disorders are often under-diagnosed and treated sub-optimally in patients with neurological disorders, which potentially leads to faster symptom progression, worse health outcomes and more frequent use of rehabilitation services (11).

The relationship between depression and anxiety and certain neurological disorders is bidirectional, i.e. patients with neurological disorders are at greater risk of developing depression and anxiety, and patients with depression and anxiety disorders are at greater risk of neurological disorders. What is of key significance in terms of the cooccurrence of mood disorders and neurological disorders is that these conditions share common neurobiological underpinnings, i.e. common neural pathways and structural and functional changes in the central nervous system (12). Genetic and environmental factors are involved in the aetiology of emotional disorders as well (13). Anxiety may be a symptom of or a reaction to a neurological disorder. However, it may also be a side effect of the medications used or a manifestation of a comorbidity (12).

Depression is the most common mental disorder in neurological patients, with average prevalence rates of between 25 and 40% (10). It may occur in patients with conditions such as epilepsy, migraine, neurodegenerative disease, multiple sclerosis and Parkinson’s disease (14). Particular attention should be paid to the issue of post-stroke depression. A recent meta-analysis based on data from studies including more than 20,000 patients reported a frequency of depression of approximately 30% in the first year after stroke (15). Post-stroke depression has a negative impact on rehabilitation outcomes, which results in chronic disability, reduced quality of life and increased mortality (16, 17). Comorbid depression in patients with neurological conditions is also associated with a poorer course of neurological illness and greater risk of the patient failing to respond to neurological therapies (12). Other common mental disorders in stroke survivors are anxiety disorders. However, studies dealing with the impact of anxiety on the course of neurological disorders and the functional status of neurological patients are very limited. It is estimated that anxiety affects approximately 25% of stroke patients (18) and is a significant determinant of the overall quality of life of these patients (19, 20).

Given the significant role of psychological variables in the persistence of disability in patients with neurological disorders, the main aim of the study was to assess to what extent the overall performance status of these patients and their satisfaction with life are affected by emotional factors, i.e. levels of depression and trait anxiety.

## 2 Materials and methods

### 2.1 Study design

Data of this a Cross-Sectional Cohort Study were collected as part of research carried out under the scientific project titled “Adherence as the responsibility of pre-seniors and seniors in the therapeutic process”, whose main aim was to assess the level of self-awareness relating to health and treatment among individuals aged over 55. The research included primary health care centres and the Universities of the Third Age in Płock. The project was carried out between January and November 2022.

### 2.2 Study population

The study was conducted using a diagnostic survey and clinimetric scales. The inclusion criteria were as follows: residency or domicile in Płock, age over 55 years, absence of cognitive impairment. Once voluntary informed consent had been obtained from the individuals enrolled into the study, the participants completed the survey in paper form or electronically on the Limesurvey platform. A total of 2,040 individuals took part in the project. Of these, a cohort of 229 patients with neurological disorders was selected. The patients studied were divided into two groups, i.e. stroke survivors and patients with other neurological disorders, to assess potential inter-group differences. Ultimately, 80 stroke patients and 149 patients with other neurological disorders were included in the study.

### 2.3 Methods

The following selected demographic variables were analysed: sex, age and education level. Psychological factors were assessed using the following standardised psychological instruments:

The level of depressive symptoms was assessed using the Polish Version of Beck Depression Inventory (BDI) (21).

The BDI contains 21 groups of statements assessing the intensity of de-pressive symptoms on a scale from 0 to 3. Respondents are asked to choose the one statement in each group that best describes how they have been feeling over the period indicated. The depression severity total score is calculated by adding up the scores for all the 21 items (22). The following score ranges were used: 0 – 11 – not depressed, 12 – 19 – mild depression, 20 – 25 – moderate depression, 26 or more – severe depression.

The level of trait anxiety was measured using the Trait Anxiety Scale (Pol. Skala Lęku - Cecha, SL-C). It contains 15 items, for which the stem question is “How of-ten do you experience the following…”. The items are rated on a 4-point scale from “often” to “never”. Responses are scored from 0 to 3. The total score is calculated by adding all item scores and ranges between 0 (minimum level of trait anxiety) and 45 (maximum level of trait anxiety) (23). Raw scores were converted to sten scores. The following score ranges were used: Sten scores of 1– 4 – low scores, Sten scores of 5 – 6 – medium scores, Sten scores of 7 – 10 – high scores.

The level of satisfaction with life was assessed using the Satisfaction with Life Scale (SWLS) (24). The instrument contains 5 statements. Respondents are asked to think about their whole lives and rate their agreement with each item on a seven-point Likert scale. The response scale is as follows: 1 – strongly disagree, 2 – disagree, 3 – slightly disagree, 4 – neither agree nor disagree, 5 – slightly agree, 6 – agree, 7 – strongly agree. The possible score range is 5–35. The higher the score, the higher the satisfaction with life (25). Raw scores were converted to sten scores. The following score ranges were used: Sten scores of 1– 4 – low scores, Sten scores of 5 – 6 – medium scores, Sten scores of 7 – 10 – high scores.

The overall performance status of study participants was assessed using the ECOG (Eastern Cooperative Oncology Group) Performance Status Scale (26). The scale ranges from 0 to 5, where: 0 = fully active, able to carry on all pre-disease performance without restriction, 1 = restricted in physically strenuous activity but ambulatory and able to carry out work of a light or sedentary nature, e.g., light house work, office work, 2 = ambulatory and capable of all self-care but unable to carry out any work activities; up and about more than 50% of waking hours, 3 = capable of only limited self-care; confined to bed or chair more than 50% of waking hours, 4 = completely disabled; cannot carry on any self-care; totally confined to bed or chair, 5 = dead (26)

### 2.4 Ethical considerations

The study was carried out following the recommendations and was reviewed and approved by the Bioethics Committee of the Mazovian Academy in Płock (Statute No. KB/N/BN/P/1.2021 dated March 15, 2021). All subjects gave written informed consent in accordance with the Declaration of Helsinki.

### 2.5 Statistical analysis

In our descriptive analysis, we used frequency tables with response percentages. The data are presented graphically using vertical bar charts and box and whisker plots. Quantitative variables were reported using arithmetic means and standard deviations, confidence intervals, medians and quartiles. The correlation between two variables was assessed using the Spearman’s R correlation coefficient. Multiple regression analysis for a prediction model was then carried out. Differences between two or more groups were assessed using the Chi-square test for contingency tables. Statistical significance was set at p ≤ 0.05. All calculations were performed using the Statistica soft-ware (version 10.0) (StatSoft Poland), the PQStat software and Microsoft Excel spread-sheet, using the standard functions of the software.

## 3 Results

### 3.1 Demographic characteristics

The vast majority of study participants were women (71.2%). The proportion of women was higher among patients with other neurological disorders (74.5%) than among stroke survivors (65.0%). The mean age of participants was 68.6 years (SD=8.8). There were significant differences in the mean age between the two groups studied (x2 = 20.476, p=0.001). Stroke patients were significantly older (M=72.6 years) than patients with other neurological disorders (M=66.5 years). The largest proportion of participants were aged between 60 and 75 (51.5%), whereas the smallest proportion of participants were aged over 90 (1.3%). The age trend was similar for both groups. The largest proportions of participants had vocational education (32.8%) or secondary/post-secondary education (32.3%), whereas the smallest proportion of participants had primary education (16.2%). Significant differences in education level were found between the two groups studied (x2 = 15.129, p=0.002). The ‘stroke’ group was significantly more heterogonous in terms of education – it had higher proportions of individuals with tertiary education (27.5%) and primary education (22.5%) compared to the ‘Other’ group. The largest proportions of patients in the ‘Other’ group had secondary/post-secondary (39.6%) or vocational education (33.6%), whereas the smallest proportion of these patients had primary education (12.8%) (**Table 1**).

**Table 1:** Demographic characteristics of study participants.

Neurological disorders	Stroke		Other		Total	
	n	%	n	%	n	%
Sex						
female	52	65.0	111	74.5	163	71.2
male	28	35.0	38	25.5	66	28.8
Age group						
under 60	6	7.5	43	28.9	49	21.4
60-75	41	51.3	77	51.7	118	51.5
75-90	31	38.8	28	18.8	59	25.8
over 90	2	2.5	1	0.7	3	1.3
Education						
primary	18	22.5	19	12.8	37	16.2
vocational	25	31.3	50	33.6	75	32.8
secondary/post-secondary	15	18.8	59	39.6	74	32.3
tertiary	22	27.5	21	14.1	43	18.8

### 3.2 Severity of depressive symptoms (BDI)

The mean score on the BDI was 14.52 (SD=10.59). Stroke patients had a higher mean score (M=17.06) compared to patients in the ‘Other’ group (M=13.15). The minimum score (Min.) for both groups was 0. The maximum score was significantly higher for stroke survivors (Max. = 53) (**Table 2**).

**Table 2:** Mean differences in scores on the BDI.

Neurological disorders	n	M	SD	Min.	Max.	Q25	Me	Q75
Stroke	80	17.06	± 11.964	0.0	53.0	8.0	16.5	21.5
Other	149	13.15	± 9.538	0.0	43.0	6.0	12.0	18.0
Total	229	14.52	± 10.590	0.0	53.0	7.0	13.0	20.0

Most study participants did not have depressive symptoms (41.5%) or had mild depression (32.8%). Compared to patients in the ‘Other’ group, stroke patients were significantly more likely to report mild depression (33.8% *vs* 32.2%), moderate depression (20% *vs* 8.7%) and severe depression (16.3% *vs* 11.4%). The statistical significance of differences between the groups was confirmed by the significant result of the Chi-square test (x^2^ = 10.103, p=0.018) (**Table 3**).

**Table 3:** Differences in depression severity between groups.

Neurological disorders	Stroke		Other		Total	
**Severity of depression**	n	%	n	%	n	%
Not depressed	24	30.0	71	47.7	95	41.5
Mild depression	27	33.8	48	32.2	75	32.8
Moderate depression	16	20.0	13	8.7	29	12.7
Severe depression	13	16.2	17	11.4	30	13.1

### 3.3 Relationships between the level of depression and demographic factors

We found a relationship between the severity of depressive symptoms and sex. Female participants experienced higher levels of depressive symptoms (M=15.05 *vs* M=13.2) (x^2^ = 8.734; p = 0.033). Severe depression was reported by 16.6% of female patients, compared to 4.5% of male patients. Men were more likely to report mild or moderate depressive symptoms (**Table 4**).

**Table 4:** Sex differences in the severity of depression.

Sex	Female		Male	
**Severity of depression**	n	%	n	%
Not depressed	69	42.3	26	39.4
Mild depression	49	30.1	26	39.4
Moderate depression	18	11.0	11	16.7
Severe depression	27	16.6	3	4.5

We found a relationship between the severity of depressive symptoms and education level (rho=0.226, *p*=0.001). Individuals with primary education experienced the highest levels of depression – 81% of them reported depressive symptoms, with 24.3% of patients with this level of education reporting severe depressive symptoms. The majority of patients with secondary or tertiary education (51.4% and 48.8%) did not have depressive symptoms (**Table 5**).

**Table 5:** Differences in the severity of depression according to education level.

Education	Primary		Vocational		Secondary		Tertiary	
**Severity of depression**	n	%	n	%	n	%	n	%
Not depressed	7	18.9	29	38.7	38	51.4	21	48.8
Mild depression	16	43.2	21	28.0	24	32.4	14	32.6
Moderate depression	5	13.5	14	18.7	6	8.1	4	9.3
Severe depression	9	24.3	11	14.7	6	8.1	4	9.3

A statistically significant low correlation was found between the severity of depressive symptoms and age (rho=0.224, *p*=0.001). The older the participants, the more severe their depressive symptoms.

### 3.4 Level of trait anxiety - SL-C

**Figure 1:**
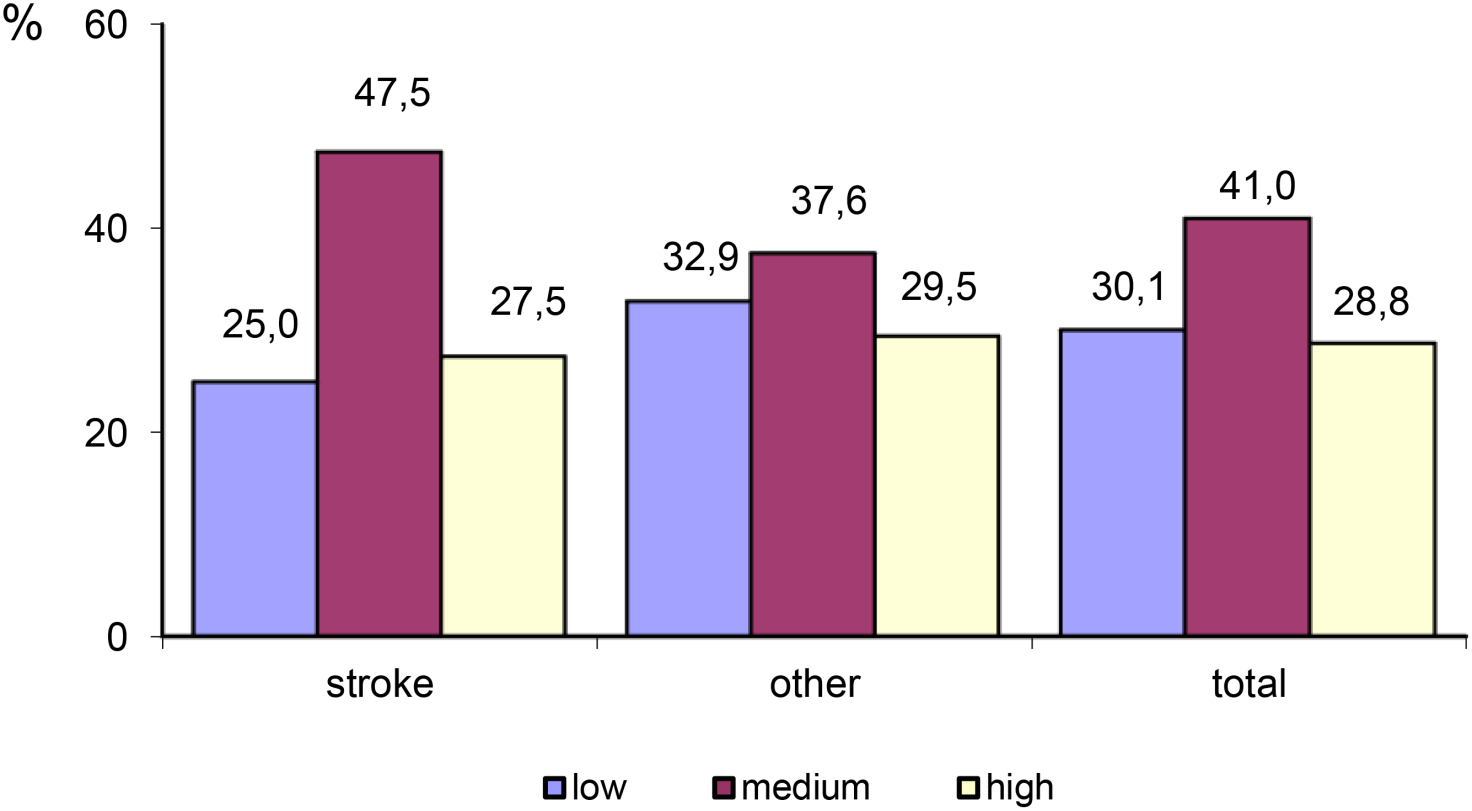
Differences in the level of trait anxiety between stroke patients and patients with other neurological disorders.

The largest proportion of participants self-reported a medium level of trait anxiety (41%), whereas the smallest proportion of participants self-reported a high level of trait anxiety (28.8%). Stroke survivors were more likely to display trait anxiety (75%) compared to patients with other neurological disorders (67.1%). Patients in the ‘Other’ group were more likely to self-report a high level of trait anxiety (29.5%) (**Table 6**; **Figure 1**).

**Table 6:** Level of trait anxiety in two groups of patients with neurological disorders and in the whole cohort studied.

Neurological disorders	Stroke		Other		Total	
**Scores**	n	%	n	%	n	%
Low	20	25.0	49	32.9	69	30.1
Medium	38	47.5	56	37.6	94	41.0
High	22	27.5	44	29.5	66	28.8

### 3.5 Relationships between the level of anxiety and demographic factors

An analysis of mean scores for trait anxiety showed no statistically significant differences between the two groups studied (x^2^ = 2.396, *p*=0.302). No significant differences were also found in the level of trait anxiety between male and female participants (x^2^ = 2.396, p=0.302) and no significant relationship was found between the level of trait anxiety and age (rho=0.066, *p*=0.317) and education level (rho=-1.738; *p*=0.374).

**Figure 2:**
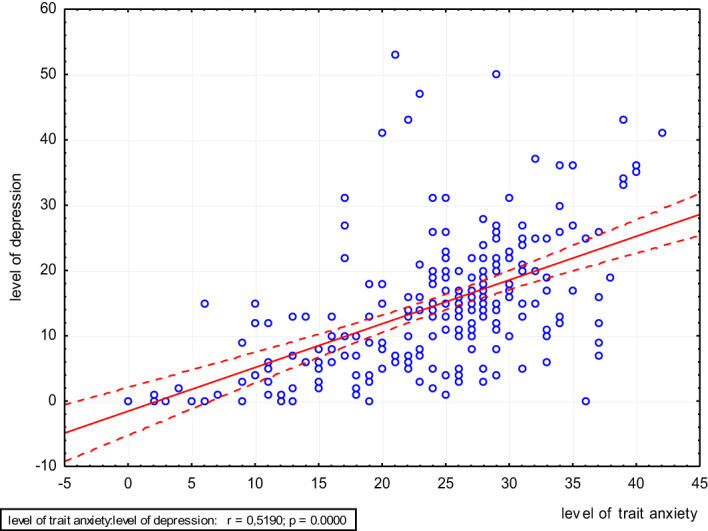
Scatter plot of the level of depression and level of trait anxiety.

A significant positive relationship was found between the level of trait anxiety and the level of depression. The higher the level of trait anxiety, the higher the level of depression (**Figure 2**).

### 3.6 Level of satisfaction with life

**Figure 3:**
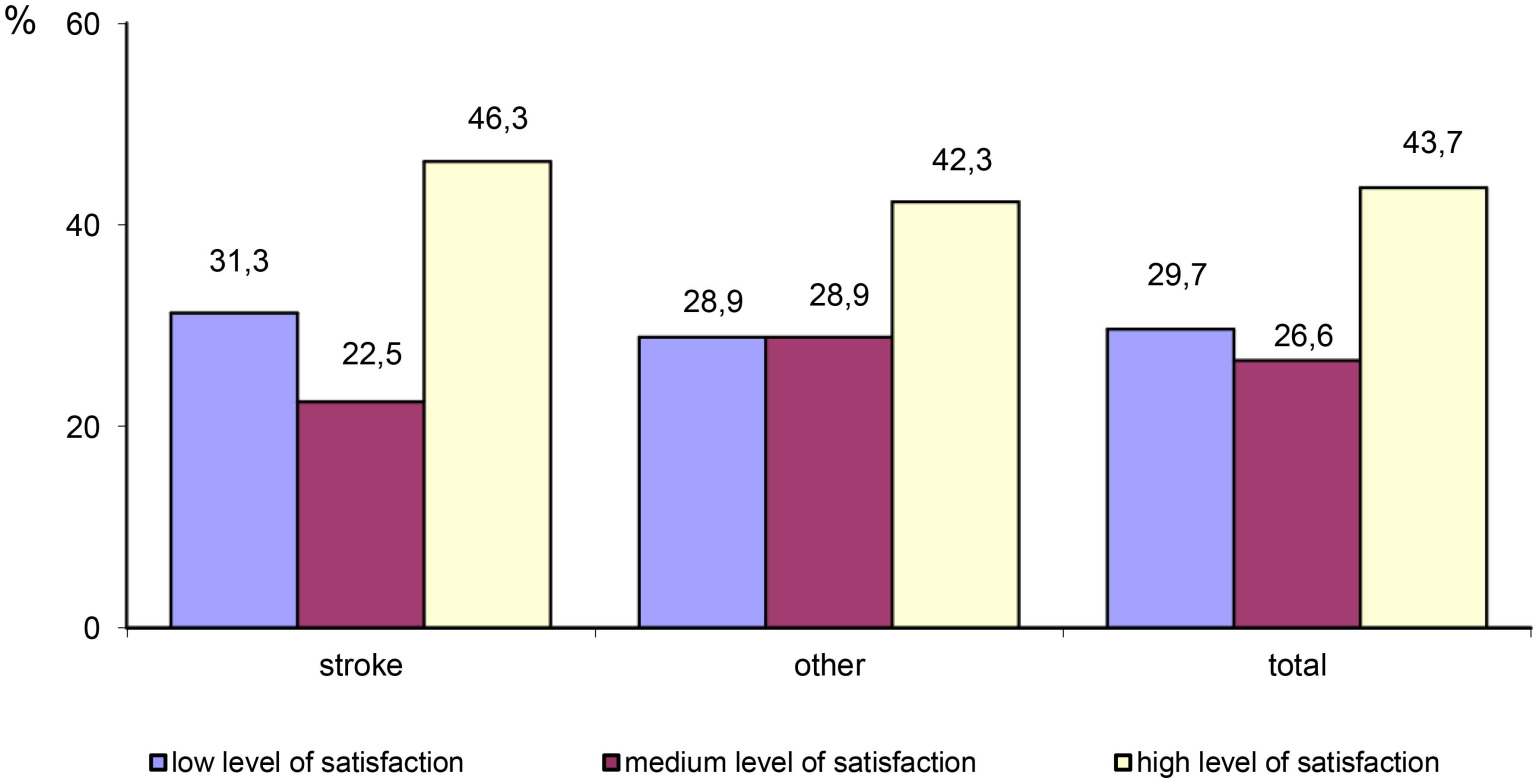
Differences in the level of satisfaction with life between stroke patients and patients with other neurological disorders.

The majority of participants (43.7%) reported a high level of satisfaction with life. A total of 29.7% of participants reported a low level of satisfaction with life, and 26.6% reported a medium level of satisfaction with life. Stroke survivors were more likely to report a low level of satisfaction with life compared with patients with other neurological disorders. However, the differences were not statistically significant (x^2^ = 1.078, *p*=0.577) (**Table 7**; **Figure 3**).

**Table 7:** Level of satisfaction with life in two groups of patients with neurological disorders and in the whole cohort studied.

Neurological disorders	Stroke		Other		Total	
**Results**	n	%	n	%	n	%
Low level of satisfaction	25	31.3	43	28.9	68	29.7
Medium level of satisfaction	18	22.5	43	28.9	61	26.6
High level of satisfaction	37	46.3	63	42.3	100	43.7

A statistically significant difference was found between men and women in the mean level of satisfaction with life (x^2^ = 32.411, p=0.001). Men had a higher mean SWLS score (25.14) compared to women (19.69). A high level of satisfaction with life was reported by 71.2% of male participants and 32.5% of female participants (**Table 8**).

**Table 8:** Level of satisfaction with life according to sex.

Sex	M	SD	Me	
Female	19.69	6.85	20	
Male	25.14	5.05	25	
Sex	Female		Male	
**Level of satisfaction with life**	n	%	n	%
Low	63	38.7	5	7.6
Medium	47	28.8	14	21.2
High	53	32.5	47	71.2

The highest mean SWLS scores were reported by participants with tertiary education (M=22.63) and those with secondary education (M=21.92). Participants with primary education had the lowest mean SWLS score (M=17.59). A statistically significant low correlation was found between education level and SWLS scores. Tertiary education was found to be associated with a higher level of satisfaction with life (rho=0.181; p=0.006) (**Table 9**).

**Table 9:** Level of satisfaction with life according to education level.

Education	M	SD	Me
Primary	17.59	5.88	17.00
Vocational	21.63	7.17	24.00
Secondary	21.92	6.81	22.50
Tertiary	22.63	6.18	23.00

No significant relationship was found between the level of satisfaction with life and age (rho=0.066, p=0.320).

### 3.6 Level of satisfaction with life according to the level of trait anxiety and level of depression

**Figure 4:**
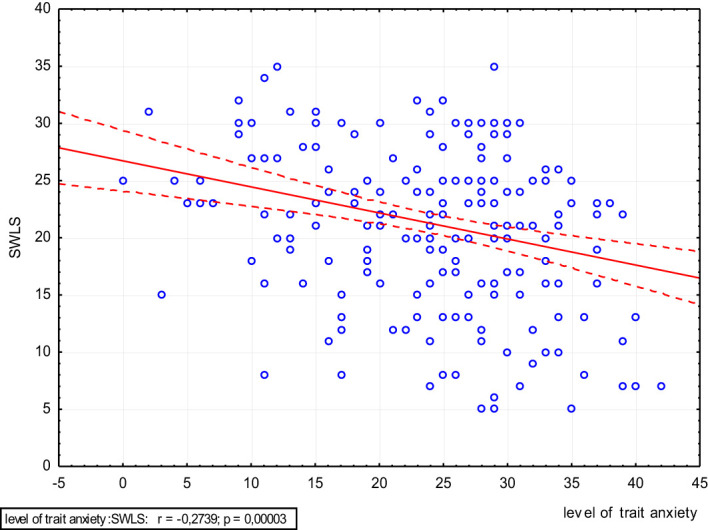
Scatter plot of satisfaction with life and level of trait anxiety.

**Figure 5:**
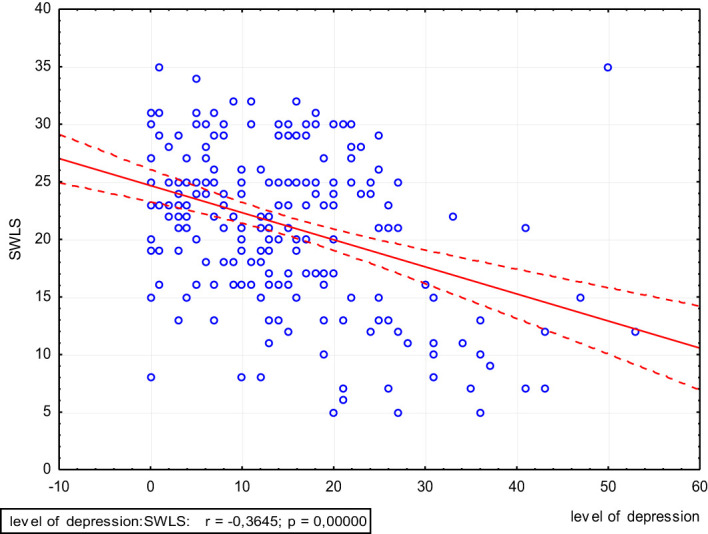
Scatter plot of satisfaction with life and level of depression.

The higher the scores on the trait anxiety scale and the BDI, the significantly lower is the level of satisfaction with life (p<0.05) (**Figures 4**, **5**).

### 3.7 Performance status (ECOG scale)

The largest proportions of participants scored 1 (“restricted in physically strenuous activity but ambulatory and able to carry out work of a light or sedentary nature”) (37.6%) or 0 (“fully active, able to carry on all pre-disease performance without restriction”) (31.9%) on the ECOG measure of performance status. The smallest proportion of participants scored 4 (“completely disabled; cannot carry on any self-care; totally confined to bed or chair”) (3.9%) on the ECOG scale. A statistically significant difference in performance status was found between stroke survivors and patients with other neurological disorders (x^2^ = 29.675, p=0.001). Stroke patients had poorer performance status. They were more likely to be unable to carry out any work activities (2) or capable of only limited self-care (3) (35% *vs* 12.1%; 10% *vs* 4.7%) (**Table 10**).

**Table 10:** Performance status in two groups of patients with neurological disorders and in the whole cohort studied.

Neurological disorders		Stroke		Other		Total	
Performance status	Definition	n	%	n	%	n	%
0	fully active, able to carry on all pre-disease performance without restriction	14	17.5	59	39.6	73	31.9
1	restricted in physically strenuous activity but ambulatory and able to carry out work of a light or sedentary nature, e.g., light house work, office work	24	30.0	62	41.6	86	37.6
2	ambulatory and capable of all self-care but unable to carry out any work activities; up and about more than 50% of waking hours	28	35.0	18	12.1	46	20.1
3	capable of only limited self-care; confined to bed or chair more than 50% of waking hours	8	10.0	7	4.7	15	6.6
4	completely disabled; cannot carry on any self-care; totally confined to bed or chair	6	7.5	3	2.0	9	3.9

### 3.8 Performance status according to demographic factors

A statistically significant low correlation was found between age and ECOG scores (rho=0.252, *p*=0.000). The performance status of patients deteriorated with age. A significant relationship was also found between performance status and education level. Tertiary education was found to be associated with better ECOG scores (rho=-0.182, *p*=0.006).

### 3.9 Predictors of the level of satisfaction with life

In order to identify the determinants of the level of satisfaction with life, univariate tests for significance were carried out. The following potential predictors were used: level of depression (BDI), level of trait anxiety (SL-C) and performance status (ECOG scale). One significant predictor was identified – the level of depression. The coefficient of multiple determination (R^2^ = 0.152, p=0.000) indicates that approximately 15% of the variation in the level of satisfaction with life can be explained by the ‘depression’ variable (**Table 11**).

**Table 11:** Predictors of the level of satisfaction with life.

Effect	SS	df	MS	F	p
Intercept	14727.95	1	14727.95	366.821	<0.001
Level of depression	803.33	1	803.33	20.008	<0.001***
Level of trait anxiety	75.47	1	75.47	1.880	0.172
ECOG	96.60	1	96.60	2.406	0.122
Error	9033.81	225	40.15		

*** p<0.001.

### 3.10 Predictors of performance status (ECOG scale)

Univariate tests for significance were also performed for the ECOG performance status variable. The following two potential predictors were used: level of depression (BDI) and level of trait anxiety (SL-C). Both the variables were found to be significant predictors of performance status. The coefficient of multiple determination (R2 = 0.239, p=0.000) indicates that approximately 23% of the variation in performance status can be explained by the two variables. However, the ECOG performance score was statistically significantly more influenced by the level of depression than the level of trait anxiety (**Table 12**).

**Table 12:** Predictors of performance status (ECOG scale).

Effect	SS	df	MS	F	p
Intercept	16.116	1	16.116	18.379	<0.001***
Level of depression	53.802	1	53.802	61.358	<0.001***
Level of trait anxiety	4.314	1	4.314	4.919	0.028*
Error	198.170	226	0.877		

* p<0.05, *** p<0.001.

## 4 Discussion

The findings from the present study show that stroke is a significant risk factor for depressive mood disorders. Depressive symptoms were reported by as many as 70% of stroke survivors included in our study, compared to 52.3% of patients with other neurological disorders. Moreover, stroke patients were more likely to report moder-ate (20%) or severe (16.3%) depression. The data are consistent with findings from other studies showing that the prevalence of depression in stroke survivors ranges be-tween 19 and 44% (27). Ellis et al. (2010) noted that depression affects approximately 30% of all stroke survivors (28). Differences in the reported prevalence rates of post-stroke depression are most likely due to different criteria used for assessing and diagnosing depressive symptoms and the use of different methods for selecting study participants, e.g. the exclusion of patients with aphasia (24). Our study also did not include patients with communication barriers, which may have had a significant impact on our reported prevalence of depression, especially given that patients with aphasia are at a significantly higher risk of depression (29). Another factor influencing comparisons and predictions as regards the prevalence of depression in different groups of patients with neurological disorders are differences between the groups studied – our study did not take into account such factors as the time since the stroke event or the extent of focal lesions and their location. The ‘Other’ group included patients with different neurological conditions among which depression is relatively rare. As a result, our reported prevalence of depression in this group of patients could be significantly underestimated.

The severity of depressive symptoms in our study participants was associated with such demographic factors as age, sex and education level. The present study included middle-aged and older individuals, i.e. those aged over 55. We found a positive relationship between age and depression severity (rho=0.224, p=0.001). Sibille and Wolkowitz et al. noted that neural networks and biological mechanisms which underlie mood regulation may be particularly affected in the case of brain ageing and neurodegenerative, inflammatory and metabolic disorders, which pose a substantial risk for older adult individuals (30, 31). Women are twice more likely to be diagnosed with depression than men (28). Female participants in our study scored on aver-age two points higher on the BDI compared with male participants and were more likely to report severe depressive symptoms. This is consistent with findings from a study by Yang et al., who noted that there are significant differences be-tween male and female patients with depression in morbidity, suicide rate and clinical symptoms (32). The higher prevalence of depression among women does not seem to be due to different rates of reported stressful life events or greater sensitivity to their pathogenic effect. Yang et al. noted in their study that the difference be-tween women and men in the prevalence of depression may be due to the development of the brain as well as its response to stress and neurotoxic substances (32). An-other determinant of higher scores on the DBI in our study was a low level of education (rho=0.226, p=0.001). Findings from a number of previous studies confirm that individuals with a low level of education have a significantly higher prevalence of depression (33). They often have lower socioeconomic status and thus face difficulties in access to specialist medical care and show poorer response to treatment.

Another factor analysed in the present study was trait anxiety. The largest proportion of participants reported a medium level of trait anxiety. Stroke patients displayed trait anxiety more frequently than patients with other neurological disorders. In their meta-analysis and systematic review of the prevalence of anxiety after stroke, Campbell Burton et al. reported that anxiety symptoms occurred in approximately 20-25% of the patients included in the studies analysed at any time after stroke (34). Poor health habits may be an indirect cause of the association between anxiety and the risk of stroke. Chronic anxiety can have serious biological effects. For example, it can lead to excess activation of the hypothalamic-pituitary-adrenal axis or arrhythmia, leading to the development of cardiovascular disease and thus increased risk of stroke (35). In the present study, we found no relationship between the level of trait anxiety and the demographic factors analysed, which in turn were found to have an in-fluence on the severity of depression. Sex-related differences in the level of anxiety may be due to differences in emotional regulation, coping styles and the use of different methods for measuring anxiety (36).

In our study, the presence of trait anxiety was found to be associated with greater severity of depressive symptoms. This means that the coexistence of anxiety and de-pression may significantly worsen prognosis and result in the secondary exacerbation of depressive symptoms (37). Depression is associated with difficulties in returning to the activities of daily living (38), worse physical functional ability (39) and persistence of cognitive dysfunction, both in the short and in the long term. Anxiety often mani-fests itself in poor self-control, a decrease in motivation and a constant feeling of fatigue (40). Thus, the coexistence of anxiety and depressive symptoms may have a maladaptive effect on the course and effectiveness of rehabilitation and significantly impair the patient’s quality of life, as well as notably affect the quality of life in patients with other neurological conditions such as multiple sclerosis (36, 41). The results of our analysis are consistent with these observations. In our study, both depression and the presence of trait anxiety were determinants of poorer ECOG performance status. This is of particular relevance for stroke patients, as it was them who had significantly poorer ECOG performance scores, indicating the persistence of disability and inability to carry out work activities. Depression turned out to be a predictor of performance status in our study participants. Moreover, it explained approximately 15% of the variation in satisfaction with life. These results are consistent with findings from a number of studies showing that mood and affective disorders have a negative impact on rehabilitation outcomes (16, 17, 42, 43).

### 4.1 Study limitation

This study encountered several potential limitations that could influence the outcomes. Primarily, the data were obtained through self-reporting, which may lead to biases as participants might report behaviours they perceive as socially desirable rather than their actual experiences. Furthermore, the voluntary nature of the study participation could have limited the number of data points, as maximizing respondent retention restricted the breadth of data collection. Additionally, although there were no significant lockdowns or disruptive policies during the data collection period, the context of the COVID-19 pandemic could have still impacted the results, affecting the general mood and health perceptions of participants.

### 4.2 Practical implication

This study highlights the need for specific prophylactic interventions to enhance mental health support within neurological rehabilitation for the older adult. Firstly, integrating routine psychological evaluations into standard neurological care protocols is crucial for the early detection of depressive symptoms and anxiety. These should include standardized screening tools and assessments by mental health professionals. Secondly, tailored integrated care models incorporating psychological counselling and group therapy are recommended based on patient needs. Additionally, community-based support programs are essential for providing ongoing support and reducing isolation among patients with similar conditions. Policymakers should focus on training healthcare providers to recognize and treat psychological conditions and support the integration of mental health services into primary neurological care settings. Lastly, ongoing research is necessary to evaluate the effectiveness of these interventions across diverse settings to refine and optimize care strategies.

## 5 Conclusions

Anxiety and depressive symptoms are significant determinants of performance status in patients with neurological disorders. Further research should place greater focus on the relationship between neurological disorders and mental disorders. Preventive measures involving psychological screening of patients with neurological dis-orders for risk factors of anxiety and depressive disorders will allow for correct diagnosis and appropriate treatment of these patients. Early psychological and psychiatric interventions can improve the cooperation between the patient and the rehabilitation team and increase the chance for the treatment to be fully effective. Prevention of mental disorders also minimizes the risk of potential somatic consequences of anxiety and depression, which may improve the patient’s overall health.

## Data availability statement

The raw data supporting the conclusions of this article will be made available by the authors, without undue reservation.

## Ethics statement

The study was carried out following the recommendations and was reviewed and approved by the Bioethics Committee of the Mazovian Academy in Płock (Statute No. KB/N/BN/P/1.2021 dated March 15, 2021). All subjects gave written informed consent in accordance with the Declaration of Helsinki.

## Author contributions

MG: Conceptualization, Formal analysis, Investigation, Project administration, Writing – original draft, Writing – review & editing. NP: Writing – original draft, Writing – review & editing. MH: Writing – original draft, Writing – review & editing. MK: Supervision, Writing – original draft, Writing – review & editing.
